# A physical action potential generator: design, implementation and evaluation

**DOI:** 10.3389/fnins.2015.00371

**Published:** 2015-10-20

**Authors:** Malcolm A. Latorre, Adrian D. C. Chan, Karin Wårdell

**Affiliations:** ^1^Department of Biomedical Engineering, Linköping UniversityLinköping, Sweden; ^2^Department of Systems and Computer Engineering, Carleton UniversityOttawa, ON, Canada

**Keywords:** action potential, biomedical electrode, electronic nerve model, nodes of Ranvier, ulnar nerve

## Abstract

The objective was to develop a physical action potential generator (Paxon) with the ability to generate a stable, repeatable, programmable, and physiological-like action potential. The Paxon has an equivalent of 40 nodes of Ranvier that were mimicked using resin embedded gold wires (Ø = 20 μm). These nodes were software controlled and the action potentials were initiated by a start trigger. Clinically used Ag-AgCl electrodes were coupled to the Paxon for functional testing. The Paxon's action potential parameters were tunable using a second order mathematical equation to generate physiologically relevant output, which was accomplished by varying the number of nodes involved (1–40 in incremental steps of 1) and the node drive potential (0–2.8 V in 0.7 mV steps), while keeping a fixed inter-nodal timing and test electrode configuration. A system noise floor of 0.07 ± 0.01 μV was calculated over 50 runs. A differential test electrode recorded a peak positive amplitude of 1.5 ± 0.05 mV (gain of 40x) at time 196.4 ± 0.06 ms, including a post trigger delay. The Paxon's programmable action potential like signal has the possibility to be used as a validation test platform for medical surface electrodes and their attached systems.

## Introduction

There has been an increased use of electrodes for electrophysiological signal acquisition in health care and biomedical research (Kelly et al., 2007; Pedrosa et al., 2012; Chen et al., 2013; Rieger et al., 2014). However, there are few reliable testing platforms that emulate the native physiological conditions found in biological sources. Biological tissues such as nerves, are inherently heterogeneous, and ensuring tissue culture survival throughout the testing period is challenging. Our overarching goal is therefore to develop an action potential (AP)-like generator that is loosely modeled on a segment of a single axon of a generic myelinated nerve that can ultimately serve as a test platform for electrodes without the reliance on tissues. An example would be electrodes designed to record peripheral nerve action potentials. In the present study, the physical hardware and proof-of-concept model development were explored.

Nerves in the human body typically range from sub-micron to 20 μm in diameter. Unmyelinated nerves have axon diameters less than about 2 μm. Myelinated nerves range from about 2–20 μm in diameter (Rushton, 1951). Tortora and Grabowski (2000) list the resting transmembrane potential of human axons to be in the range of −40 to −90 mV (inside to outside potential), with a typical value of −70 mV. The depolarization-repolarization time of the typical neuron is about 1 ms, with the largest nerves having a typical refractory period of 0.4 ms and the smallest up to 4 ms (Tortora and Grabowski, 2000). The axon membrane has a typical trigger threshold of −55 mV and excursion limits of −90 mV (potassium equilibrium potential) to 30 mV upper limit. Statistically, a 20 μm diameter fiber has an internode spacing of 2 mm and a propagation speed of about 120 m/s (Rushton, 1951) that results in a 16 μs increment time between activation of sequential nodes of Ranvier.

Mathematical models (McIntyre et al., 2004; Carnevale and Hines, 2006; Åström et al., 2015), and physical neuronal models (Lewis, 1968a; Roy, 1972; Andreasen et al., 1997; Rieger et al., 2014) have been described in literature. Most of these were based on Hodgkin and Huxley's (1952) formalization of the relationships between the transmembrane electric potential and the individual ionic current sources for the axonal process of a nerve. Around 1970, Lewis (1968a,b) and Roy (1972) developed electronic neuron models based on the Hodgkin and Huxley (1952) equations. These electronic nerves emulated axon propagation delays and synaptic gap interfaces. However, neither model offered a tissue-electrode interface. Instead, these developments were used to emulate nerve-to-nerve interactions for complex neuronal network investigations. The technology of the 1970s had limitations that forced these analog computer implementations to operate with parameters that were 10 times the physiological values, and limited long term signal stability. Other drawbacks that limited the usefulness of these axon mimics included difficulties in making multiple hardware elements function identically, and the long setup times for each HH parameter change. Andreasen et al. (1997) addressed some of the limitations of previous designs. They designed a physical test platform specialized for cuff electrodes that offered a possible solution as a more generalized electrode testing platform. It relied on mechanical movement for AP propagation that in turn made the reliance on dynamic mechanical stability very high. Recently, Rieger et al. (2014) presented a cuff-electrode specific design with a limited ability for spatial interactions. Therefore, to date, various physical models have been reported (Lewis, 1968a,b; Roy, 1972; Andreasen et al., 1997; Rieger et al., 2014). Several of these are candidates for surface electrode testing platforms if their mechanical or electrical design limitations can be overcome. Overall, however, most existing models still lack key requirements such as stability, repeatability, or amplitude programmability necessary for an electrode test platform.

An electrode test platform should allow the user to evaluate how the electrode and data acquisition system behave. Once electrode variability and characteristics are understood a better compensation for artifacts caused by that specific electrode type can be formulated. Such a test platform should also allow for better selection of measurements system components as well as design of electrode system units. These components include the wire leads, amplifier gain blocks, impedances (Meziane et al., 2013), and the tissue itself. A complete platform for comparative work on electrodes, and yet independent of the type of electrode being tested (e.g., ECG, EEG, EMG, needle, cuff) is needed. This involved the design of a platform that included electrodes to mimic the nodes of Ranvier, a potential source for the electrical AP voltage excursions, a timing generator for sequencing through the electronic nodes, and an interface to couple the test electrode. All combined, this is the generator of the AP output, and a test and calibration device for electrodes. A test model does not need to mimic any one type of tissue (e.g., muscle, nerve bundle, or single axonal process) as long as it meets the requirements of AP stability, repeatability, and amplitude programmability. The requirement for a model with a physiologically like “active” AP has not been met with previous designs, specifically some component is missing, either stability, repeatability, or coupling to electrodes.

In this study, we report on the Paxon's A-fiber like AP-generator performance in terms of the Paxon's ability to produce stable, repeatable, and programmable APs when physically coupled to electrodes.

## Materials and methods

### The physical action potential generator model

**Figure 1:**
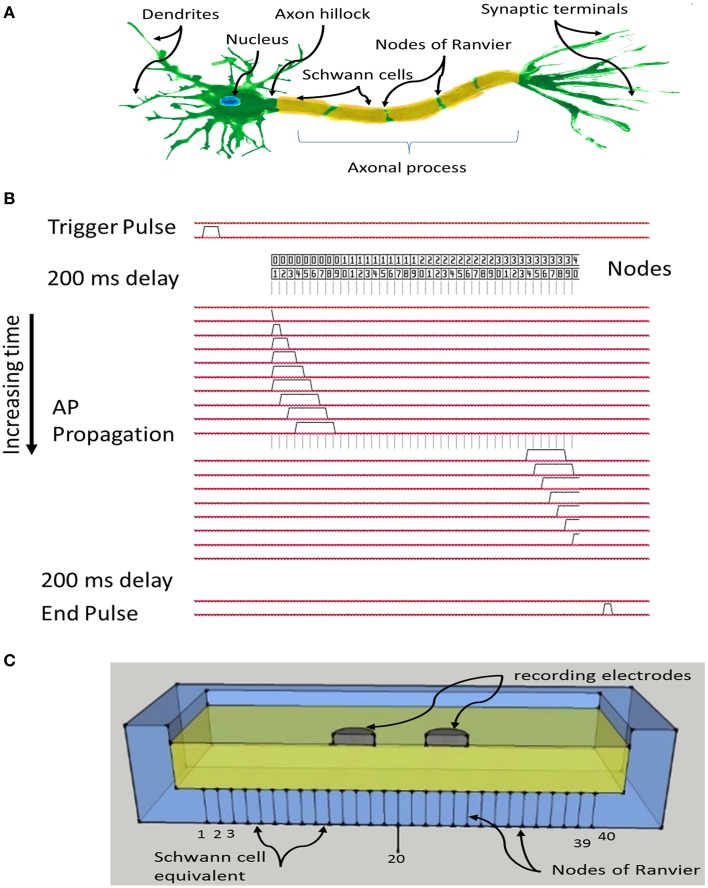
**(A)** Schematic of a myelinated nerve with labeling, region of interest is the axonal process including myelin and nodes of Ranvier; **(B)** System timing and signal propagation through nodes, 60 μs between timing lines defining Paxon propagation activity; **(C)** Sketch of test configuration, differential recording electrodes in contact with the flooded saline solution tank, positioned over Paxon nodes drawn as lines through the bottom of the tank, and centered over node 20 (of 40 nodes).

The Paxon was approximately modeled after an A-fiber, specifically a single axon of 20 μm diameter. A schematic of a myelinated neuron with the axonal section marked out is depicted in Figure 1A.

The Paxon representation of the axon was assembled with 20 μm diameter gold wire and plastic resin (EPOTek 301, Epoxy Technology Inc., USA) Figure 1C. Gold was selected over more conductive silver or copper due to its superior electromechanical stability within the testing environment. The resin acts both as the physical assembly and the inter-nodal insulator equivalent to the myelin. To limit the size, only 40 nodes of Ranvier were mimicked, each node being represented by a gold wire (Ø = 20 μm, 99.99% Au, SPM, Armonk NY, USA) and each wire was connected to a separate MOSFET electronic driver circuit which was in turn individually connected to a unique I/O pin from an ATxmega64A1 CPU (Atmel Corp., USA).

**Figure 2:**
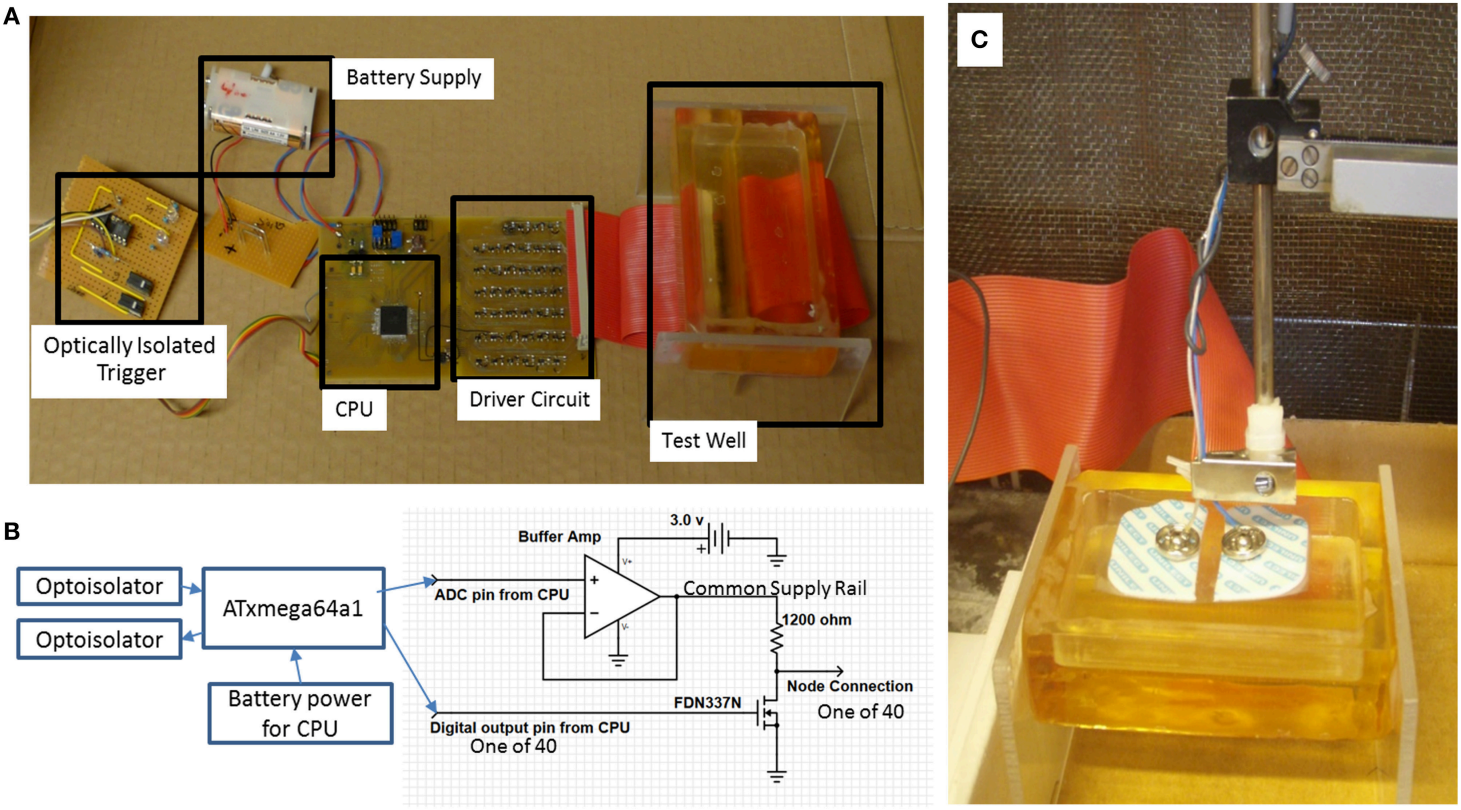
**(A)** Paxon system blocks. Battery to power the CPU and driver circuits, optically coupled galvanic isolation trigger and response to isolate Paxon in the faraday cage, CPU timing and sequencing, Driver circuit for interfacing the equivalent nodes of Ranvier, Test well assembly with resin insulated gold wire nodes. Each node is connected to an individually controlled driver circuit; **(B)** block diagram with output driver schematic; **(C)** photo of physical testing setup with standard surface Ag-AgCl electrodes in place.

The program in the CPU emulates an AP propagation by selectively turning individual I/O pins on or off at 60 μs intervals (Figure 1B), with a signal amplitude excursion of ~100 mV (Figure 2B), controlled by the driver circuit. The system was controlled by software developed in ICCV7 for AVR (ImageCraft Inc., USA) and AVR Studio 4 (Atmel Corp., USA). A NI-USB6251 data acquisition module (sampling frequency = 5 kHz, National Instruments Corporation Austin, TX, USA), and attendant LabVIEW (Version 8.2) program were used in data collection. A first order passive low pass filter with a cut-off frequency of 750 Hz was placed between the acquisition electrodes and the acquisition module to reduce unwanted environmental noise contributions. This 750 Hz filter was designed to meet the Nyquist–Shannon sampling theory constraints. The Filter attenuates the input amplitude at a rate of 20 dB per decade above a frequency of 750 Hz such that at a frequency of 2.5 kHz the input is attenuated by 50%. All data collected from the Paxon is presented with a 40x gain. Figures 2A,C presents a photo documentation of the Paxon assembly and Figure 1C the nodes of Ranvier located along the center line of the well. The wires forming the nodes pass through the bottom of the well, and are terminated at a terminal block. A flat cable connects the well terminal block to the control board. The well dimensions are; 11 × 56 × 92 mm, resulting in a maximum electrode to node displacement of 11 mm above and 28 mm laterally. The Paxon hardware and electrodes under test were housed in a Faraday cage in order to reduce induced electrical noise from the surrounding environment (e.g., wiring, oscilloscope, and local radio tower).

### Programming of an action potential

The Hodgkin and Huxley mathematical model for an AP is based on individual physiological ion flux currents across the axonal membrane. The model is defined according to Equations (1) and (2) Hodgkin and Huxley (1952).

(1) $$\begin{array}{l} \frac{d^{2}V}{dt^{2}}=k(\frac{dV}{dt}+\frac{1}{C_{M}}({\bar{g}}_{K}n^{4}\left(V-V_{K}\right)+{\bar{g}}_{Na}m^{3}h\\ \;\left(V-V_{Na}\right)+{\bar{g}}_{l}\left(V-V_{l}\right))) \end{array}$$

where:

$\frac{d^{2}V}{dt^{2}}$ =second time derivative of the voltage (V)

*V* = transmembrane voltage (V)

*V_K_*, *V_Na_*, *V_l_* = *Nernst's voltage for potassium, sodium and leakage ions respectively (V)*

${\bar{g}}_{K},\;{\bar{g}}_{Na},\;{\bar{g}}_{l}$ =Sodium, potassium and leakage conductivityper unit area $\left(\frac{\text{S}}{{\text{m}}^{2}}\right)$

*C*_*M*_ = membrane capaciatance per unit area $\left(\frac{\text{F}}{{\text{m}}^{2}}\right)$

(2) $$\begin{array}{r} \text{k}=\;\frac{2R_{2}\theta^{2}C_{M}}{a} \end{array}$$

where:

*k* = axon property constant ($\frac{\Omega \text{F}}{{\text{s}}^{2}{\text{m}}^{3}}$)

*R*_2_ = distributed resistance of axoplasm ≫ resistance of external conducting fluid ($\frac{\Omega }{\text{m}}$)

θ = propagation velocity ($\frac{\text{m}}{\text{s}}$)

*a* = cross−sectional axon area (m^2^)

From this statistically derived equation, along with a measure of the internal and external ionic concentrations and temperature, the steady state and AP transmembrane potentials can be calculated for a single point along the axonal process.

The Paxon's defining Equation (3) implement a simplified behavioral model with activity similar to that of the Hodgkin and Huxley (1952) Equations (1) and (2) consisting of a train of pulses propagating along active nodes. The Paxon generated pulse at any time τ as distributed along the nodes is defined as:

(3) $$\begin{array}{rc} P\left(\tau \right)= & \left\{\alpha \left(\tau \right),\;\alpha \left(\tau \right)+\;1,\;\ldots ,\;\beta \left(\tau \right)-\;1,\;\beta \left(\tau \right)\right\};\\ P\left(\tau \right)\subseteq \eta \end{array}$$

where:

*P*(τ) = the vector of active physical nodes

*N*_η_ = total number of nodes of Ranvier

η = set of nodes of Ranvier implemented in the well;

η = {1, 2, …, *N*_η_}

τ = discrete time, assuming a time step of 60 μs, resulting in the corresponding continuous time t = τ × 60 μs; τ *ϵ Z*

α(τ) = the node where the pulse starts at a given time τ;

α(τ) ϵ η

β(τ) = the node where the pulse ends at a given time τ;

β(τ) ϵ η; β(τ) ≥ α(τ)

As the simulation starts at node 1 and propagates toward node *N*_η_, P(τ) will contain a series of consecutive nodes that are active from node α(τ) to β(τ). The maximum width or cardinality of the pulse, |P(τ)|, is described as:

(4) $$\begin{array}{l} |P\left(\tau \right)|=\beta \left(\tau \right)-\alpha \left(\tau \right)+1 \end{array}$$

This assumes that the number of nodes (*N*_η_) is large enough that the maximum pulse width does not span all nodes (max *|P(τ)|* < *N*_η_). It is also assumed that the stimulation starts at time τ = 0, so *P(τ)* = ∅ (empty set) is true while τ < 0 or τ ≥ *[N*_η_ + *max(|P(τ)|)]*, which implies no nodes in the well area are active because the proposed active nodes are located outside of the working range of the Paxon well. Equation (3) defines the activity at the electrical nodes in both time and space (axial node position) without consideration of the amplitude. The drive potential amplitude (A) in turn is programmable by one set point for all outputs. This results in independent controllability of the internode timing, rate of propagation, and pulse excursion amplitude. The activity at the nodes form a propagating wave of controlled amplitude along the length of the physical model in a manner similar to that of the myelinated nerve AP, where each electrical node is equivalent to one node of Ranvier (Figure 2B). The Paxon signal reference is the zero volt supply line which is equivalent to the inner membrane wall potential in the Hodgkin and Huxley (1952) model (1) and (2). The axonal external environment is only an approximation of the bulk node and tissue parameters. The impedance between source and electrode is based on these bulk parameters of the intervening tissue mimic, 0.9% saline solution.

### Evaluation

#### Experimental setup

The active nodes of the Paxon were submerged in a bath of 0.9% NaCl (B. Braun Melsungen AG, Melsungen, Germany) solution that acts as the intervening tissue mimic for testing (Rieger et al., 2014). Starting an AP was accomplished by a rising edge on a CPU I/O pin. Data were collected using a pair of standard Ag-AgCl electrodes (4831Q, Unomedical a/s, Birkerød, Denmark) placed in a bipolar configuration as depicted in Figures 1C, 2C with gel removed. A bipolar test electrode was constructed from individual electrodes with pole centers spaced 21 mm apart. This bipolar electrode configuration was used for all experiments, typically centered on node 20 and axially aligned with the Paxon's equivalent nodes of Ranvier unless otherwise stated. The electrodes were suspended in a saline bath at a fixed distance of 10 mm above the Paxon's equivalent nodes of Ranvier. The Paxon, unlike a biological axon, does not have a cell membrane nor intracellular components. The saline functions as a conductive fluid coupling the electrodes being tested to the Paxon nodes in place of the intervening tissues.

The initial experimental conditions were applied to Equation (3) with a maximum pulse of width max(|*P(τ)*|) = 5, drive potential *A* = 1 V and τ = 60 μs. The trigger signal originated from the NI data acquisition system. A delay of 200 ms was applied before the emulated AP propagation was started. After the AP passed the last emulated node of Ranvier a further 200 ms delay was added after which the Paxon microcontroller sent a stop signal back to the data acquisition system. This handshaking methodology simplified post-processing data analysis.

#### Repeatability and stability

In order to verify the repeatability and consistency of the generated signal, multiple runs (*n* = 50) at one configuration setting [max(|*P(τ)*|) = 5, drive potential, *A* = 1 V and τ = 60 μs] were taken from the Paxon. For each AP the direct current offset was removed by data mean value subtraction. The mean (*m*) and standard deviation (*s.d*.) were calculated for time from start (set to 5% of the peak value) for the rise, the positive peak excursion, the negative peak excursion, and end of signal time. The *m* and *s.d*. were also calculated for the amplitude variation at resting, positive, and negative maxima. These data were used to confirm repeatability and stability of the Paxon. An inter step stability test was also performed where each node was individually tested with the same settings as above for timing variations and amplitude differences node to node. This node to node test was accomplished by connecting the negative input of the data acquisition system to the internal circuit zero. The data acquisition system positive input was directly connected to node 1, and an AP cycle was run. The connection was then made to node 2, and the cycle repeated. This process was repeated until node 40 was measured.

#### Action potential train programming and working range

The number of active nodes [max(|P(τ)|) = 0, 1, 2, 5, 10, 15], inter-nodal timing (τ = 60 μs), and drive potentials (A = 0, 0.5, 1.0, 1.5, 2.0, 2.5, 2.8 V) were adjusted, one parameter at a time (*n* = 42), to determine the influence on the final acquired AP-signal. In this model, the physical spacing of the nodes were not changing from their 2 mm spacing, regardless of the possible changes in propagating velocity. All data analyses were carried out in MATLAB (R2011b, The Mathworks, Inc., MA, USA). MATLAB's cftool was used to find the coefficients to a second order polynomial equation using linear regression. For example, one AP was recorded for max(|P(τ)|) = 15 and A = 2.8 V, then A was reduced to 2.5 V and another AP was recorded, sequentially stepping through the complete set of variables down to max(|P(τ)|) = 0 and A = 0 V.

#### Physiological recordings of ulnar nerve

**Figure 3:**
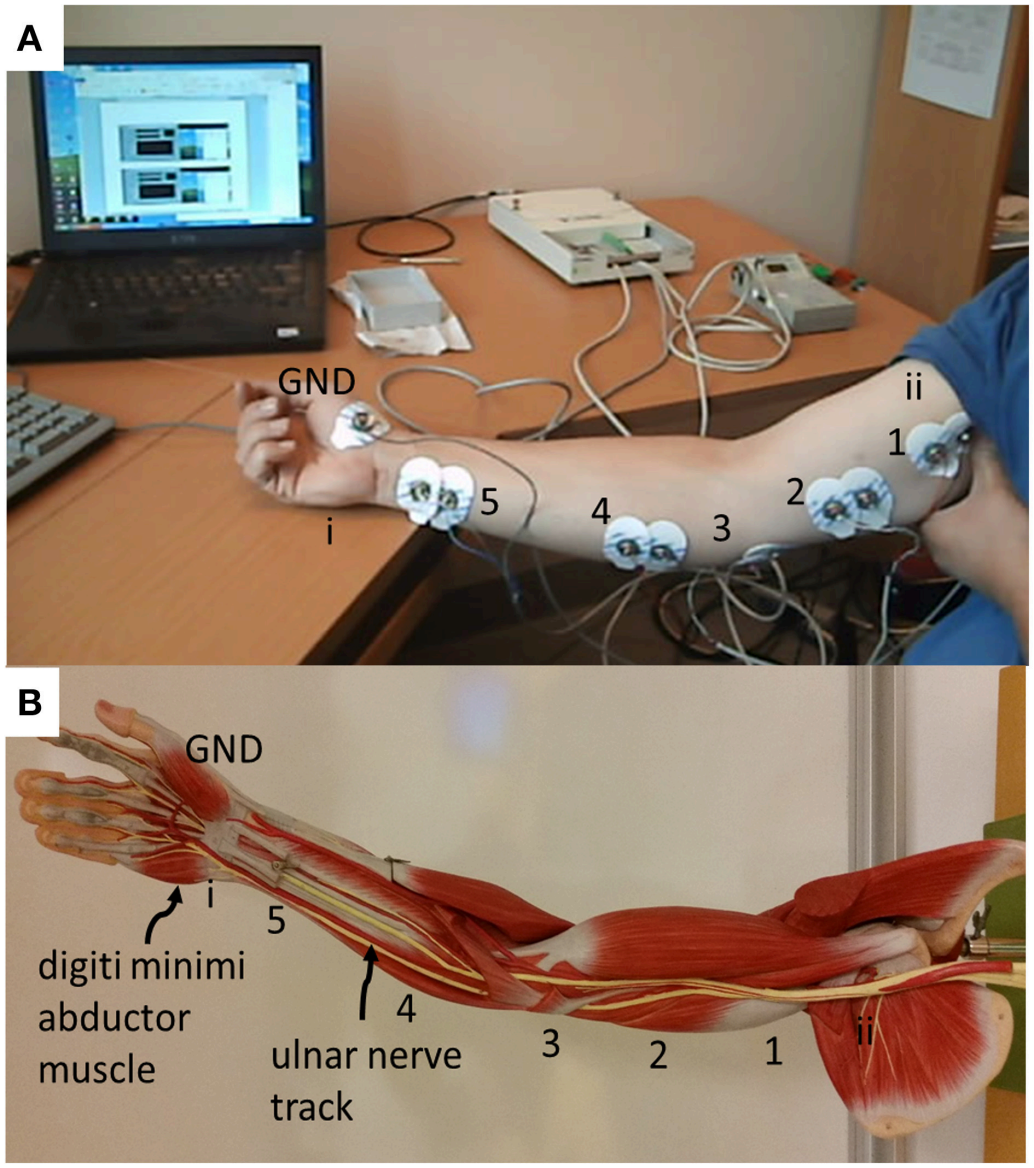
**(A)** Placement of electrodes for physiological measurement of conduction of the ulnar nerve, based on standard surface Ag-AgCl electrodes applied differentially (21 mm center-to-center spacing) located along ulnar nerve track. Center distance, 18 cm between 5 and 4, all others 8 cm apart; **(B)** photo showing the location of the ulnar nerve track and the digiti minimi abductor muscle as well as labeling of recording and triggering points.

A modified clinical protocol “Ulnar motor nerve to the digiti minimi” (Buschbacher and Prahlow, 2006) was used to verify the acquisition system as it is applied to the Paxon (Figure 2A). The measurements taken were used only as an amplitude and duration reference against which the Paxon was compared. Measurements were done on a healthy consenting volunteer (ML). The protocol modification was to measure along the ulnar nerve track at the typical stimulation points (Figure 3). The ulnar nerve was stimulated at either the upper arm entry point or before the digiti minimi abductor muscle (Figure 3, point i and ii). Stimulation was accomplished with a transdermal stimulator at 12 mA pulse repeated rate of 2 s (Digi Stim III, Norcuron, Neuro Technology, Houston, Texas, USA). Recording was performed using five sets of Ag-AgCl electrodes in bipolar configuration with a 21 mm spacing (31.1925.21, Kendall™ Arbo^*^, Neuhausen am Rheinfall, Switzerland) positioned at points 1–5 as shown in Figure 3. Recording of the physiological AP was taken at all five electrode pairs concurrently with the same configuration settings used with the Paxon. A 6th channel was used to record the stimulation event. The signal was filtered with an 8th order software notch filter with notch set between 48 and 53 Hz to remove power line noise and a 2nd order low pass filtered at 100 Hz to remove unwanted higher order noise.

## Results

The hardware and software preformed the required design tasks and were controllable by the external data acquisition system. The propagation traversed the 40 emulated nodes of Ranvier at an update time of 60 μs per step as designed.

### Repeatability and stability

**Figure 4:**
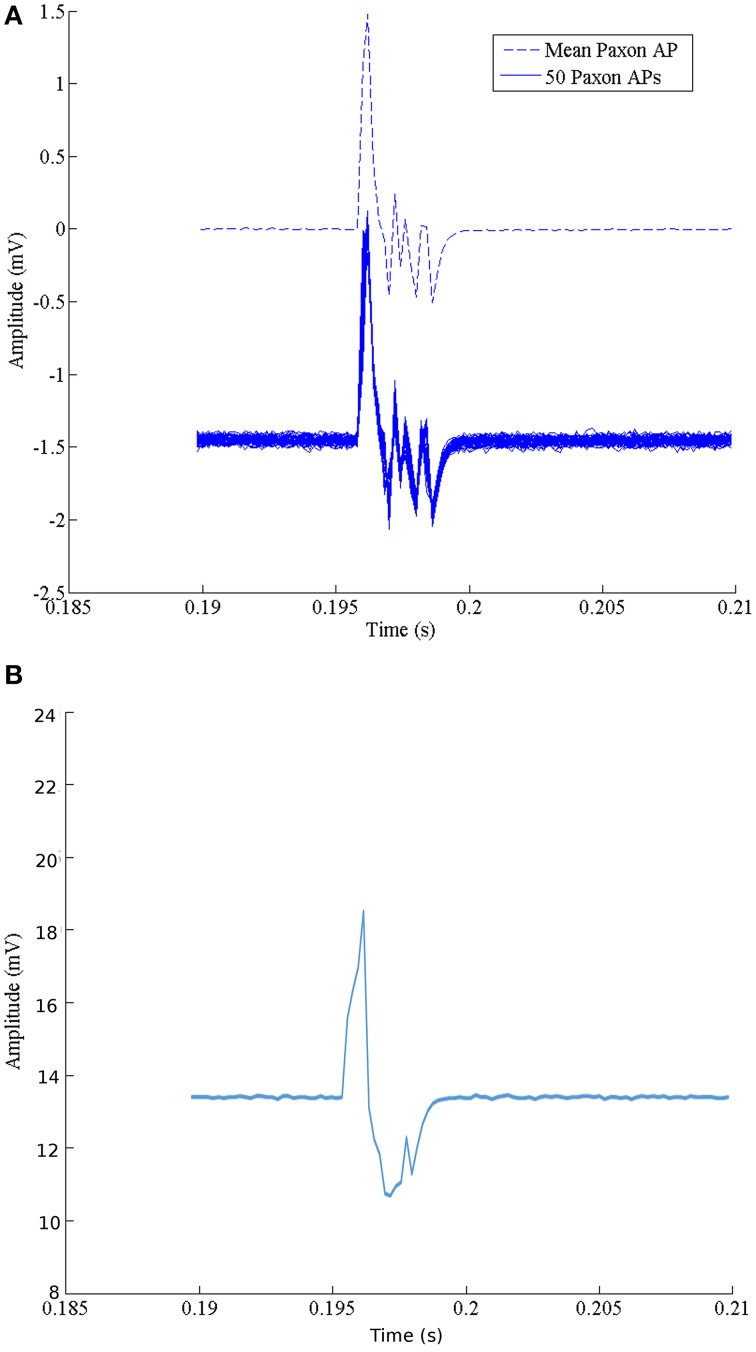
**(A)** Characteristics of the Paxon's AP event. The solid lines are a collection of 50 events using standard surface Ag-AgCl electrodes configured as a differential pair with a 21 mm center-to-center, centered over node 20, axially aligned and located 10 mm above the Paxon nodes. The dashed line is the mean average of the 50 recordings with offset removed. **(B)** Data capture of one event with glitch removed.

The set of 50 recordings at one configuration is shown in Figure 4A. The average with DC offset removed is represented with a dashed line. Table 1 gives the calculated *m* and *s.d*. of the jitter at the start of rise, peak positive excursion, peak negative excursion, and end of signal times, as well as amplitude variation at resting, positive, and negative maxima.

**Table 1:** **Comparison of multiple datasets from one paxon configuration with paxon settings of |P(τ)| (number of nodes in AP) = 5, A (Paxon drive potential) = 1 V, and τ (node update time) = 60 μs, repeated 50 times, signal gain set to 40x time is calculated from start trigger to the AP edge which is about 200 ms delay from the trigger**.

**Parameter**	**m ± s.d**.	**Minimum**	**Maximum**
Rise to 5% of peak (ms)	196.20±0.06	196.20	196.40
Peak positive excursion (ms)	196.40±0.06	196.20	196.40
Peak negative excursion (ms)	198.20±0.78	197.20	198.80
End of signal times (ms)	198.40±0.66	197.20	198.80
Positive maxima (mV)	1.50±0.06	1.40	1.60
Negative maxima (mV)	−0.52±0.03	−0.45	−0.59
Noise floor mean value (mV)	0.00280±0.00069	0.000260	0.00410

**Figure 5:**
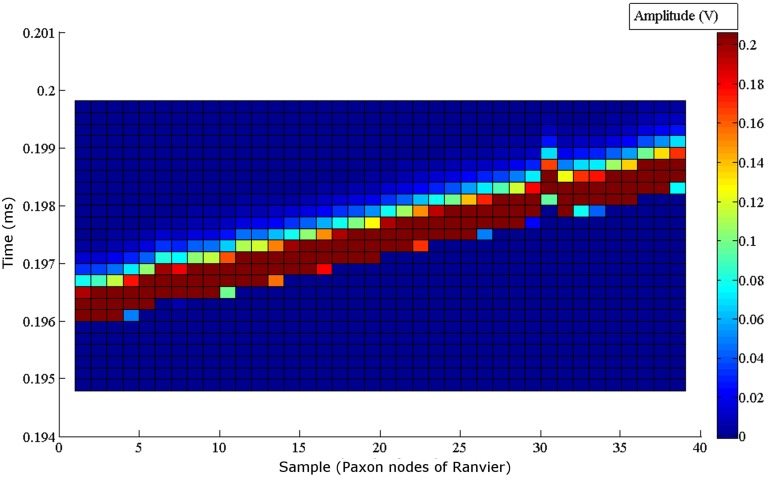
**Node to node step sequence of the Paxon nodes of Ranvier**. Each node is individually recorded. All 40 node recordings are stacked to present the step wise activity. The drive configuration of, A = 1 V drive, τ = 60 μs steps, inclusive of the 200 ms delay post trigger to AP generation, and (|P(τ)| = 5 is used. A glitch can be seen around node 32, which coincides with a coding error in the handoff between two 32 bit software registers in the microcontroller. The amplitude of the measured signal is shown with the color grading defined by the scale bar on the right.

The noise floor mean value is not zero (Table 1). Figure 5 graphically presents the data as measured from each output, node of Ranvier, one node at a time after the trigger delay. A change to a 60 μs programmed step time was needed, and the measured time was nominally 61.06 μs with a jitter of 0.59 to -1.04 μs. The pulse length for this specific test was increased to 60 μs for visualization within the Paxon data collection setup. The mean amplitude and standard deviation were 5.12 ± 0.0025 mV. There is a sequencing glitch around node 32–33 that can be seen as the misstep in the plot (Figure 5). This glitch was caused by a coding error at the handover between two 32 bit software shift registers. Figure 4B is the captured AP with the glitch repaired with A = 3.1 V, other parameters remain unchanged.

### Action potential train programming and working range

**Figure 6:**
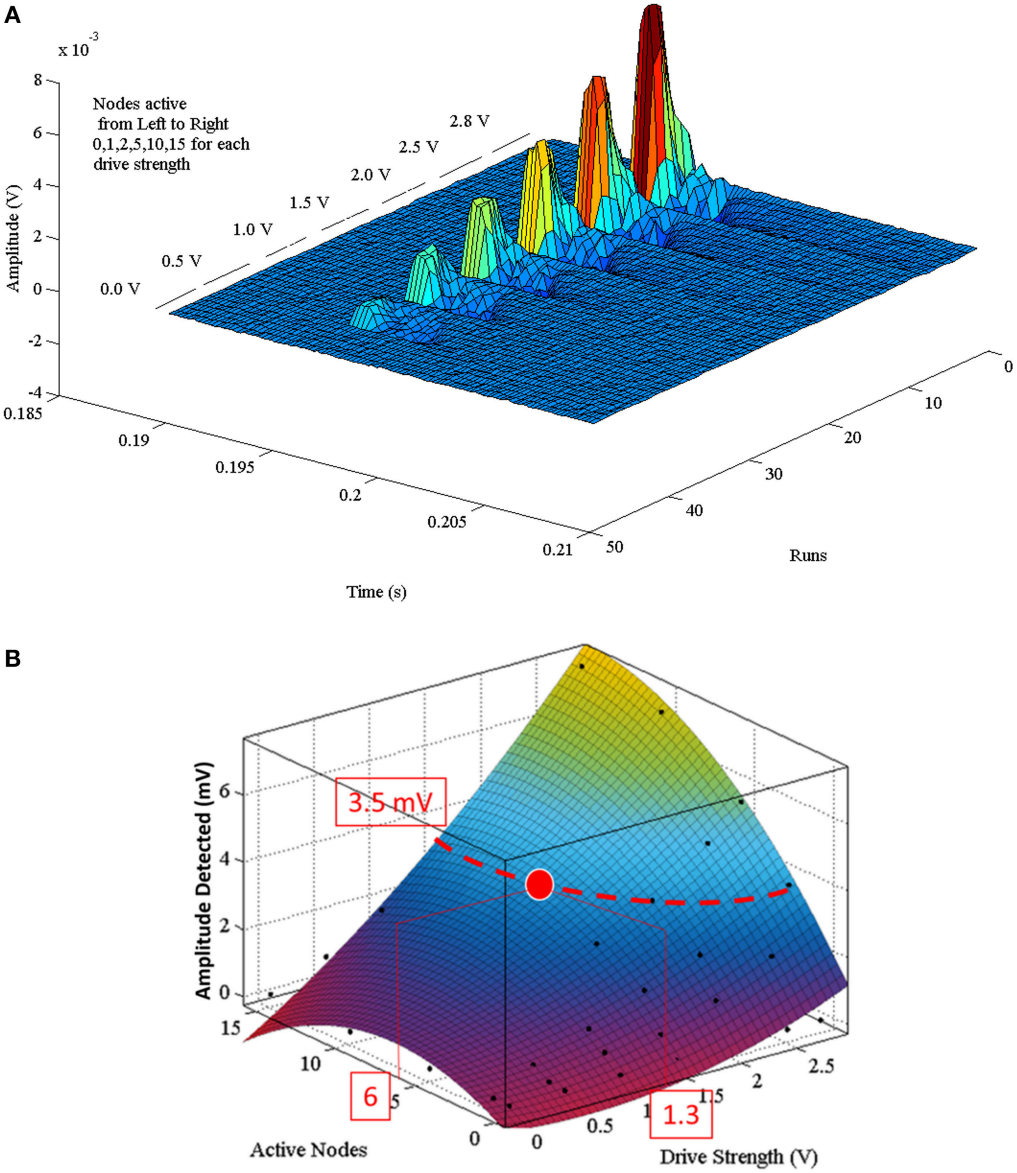
**(A)** Composite of multiple sequential Paxon's AP recordings. Each AP was recorded with a different parametric setting. Runs are the individual AP recordings. Time is in range of interest. Each group is broken into separate drive potentials (A = {0.0, 0.5, 1.0, 1.5, 2.0, 2.5, 2.8} volts). Each grouping is further subdivided by the number of active nodes (|P(τ)| = {0, 1, 2, 5, 10, 15}). **(B)** Is the programming polynomial fit surface from Equation (5). A programming example: to attain a desired detected signal peak of 3.5 mV an isopotential line is projected on the surface, then either a (|P(τ)|) or (A) value is chosen that intersects with the isopotential line. If |P(τ)| = 6 is chosen, then A must be set to 1.3 V.

A complete set of APs were generated by variation of one parameter [max(|*P(τ)|)*={0, 1, 2, 5, 10, 15} and *A* = {0, 0.5, 1.0, 1.5, 2.0, 2.5, 2.8 V}] at a time (Figure 6A). The dataset forming Figure 6A can be described by the second order polynomial (5) equation surface. Applying Equation (5) with a finer granularity step size for |*P(τ)*|and *A* results in the plot shown in Figure 6B. Equation (5) is the defining equation to extract the parameters for |*P(τ)|* or *A* given a desired detected amplitude.

(5) $$\begin{array}{r} f\left(max\left(\left|P\left(\tau \right)\right|\right),A\right)=\\ \frac{p_{00}+p_{10}N_{p}+p_{01}A+p_{20}N_{p}^{2}+p_{11}N_{p}A+p_{02}A^{2}}{4}*10^{-7} \end{array}$$

where:

max(|*P(τ))*|= maximum pulse width or cardinality {0–15} in steps of 1

A = drive strength (V) settable between 0–2.8 V in steps of 0.7 mV

*f* (max(|*P*(τ)|), *A*) = the output of the combination of *N*_p_ and A (V)

p_00_ = −221.1;*p*_10_ = −246.4;*p*_01_ = 304.9;*p*_20_ = 308.0;

*p*_11_ = 148.1;*p*_02_ = −22.9

The polynomial describing the predictive detection is valid for the configuration used, specifically 10 mm above source, aligned axially, centered over node 20, with differential electrode centers spaced 21 mm apart. A programming example is described with the red lines in (Figure 6B) for configuring a desired detection amplitude of 3.5 mV. In order to obtain that value the curve of the surface is used to find either the pulse width [max(|P(τ)|)], or the drive strength (A) that is within the generating limits of the model. Once one of these is defined, the other variable becomes fixed as well. If max(|P(τ)|) = 6 is chosen, then A must equal 1.3 V.

### Physiological recordings of ulnar nerve

**Figure 7:**
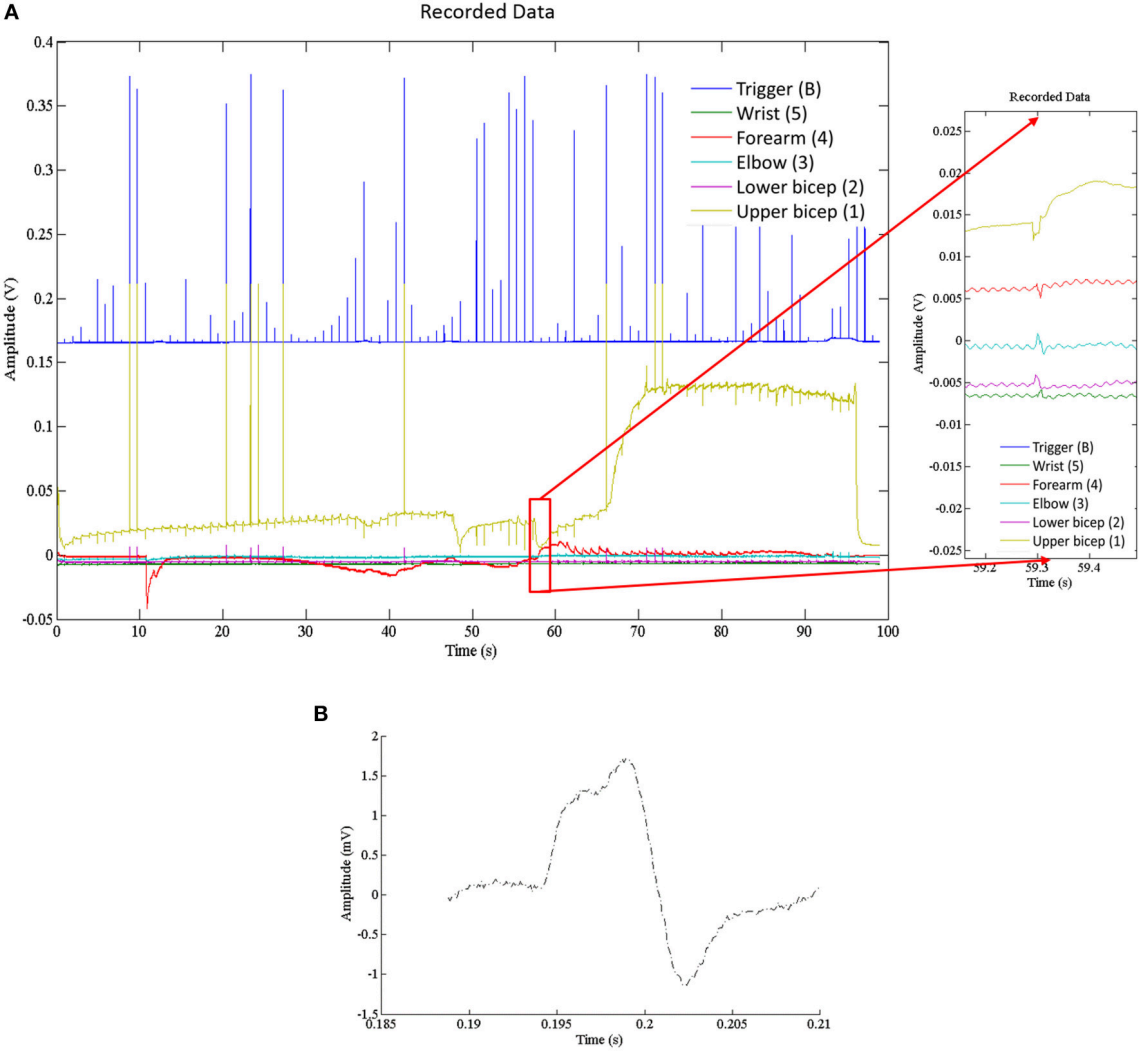
**(A)** Electrophysiological recordings of AP conduction along the ulnar nerve. Recording electrodes placed as in Figure 3. Exploded image is a magnification of one ulnar AP event. **(B)** Physiological AP recorded from ulnar nerve at the wrist electrode set (Figure 3 position 5 with stimulation at point ii).

Recorded data, Figure 7A, along the A-fiber ulnar nerve path for all five Ag-AgCl electrode pairs, were successfully obtained. The nerve stimulator signal was recorded on a 6th channel as an event trigger. One of these recorded APs from the wrist differential electrode (electrode pair 5 stimulation at point ii, shown in Figure 3) is presented in Figure 7B to show that standard surface Ag-AgCl electrodes can be used to record the AP from nerve tracks. These physiological recordings are not single axon events but compound action potentials from the nerve bundle that is externally stimulated. These physiological AP recordings are comparable to the APs recorded from the Paxon with settings of max(|P(τ)|) = 5, A = 1 V. There are similarities in both amplitude and duration of the detected AP (Figure 4A, mean Paxon AP, and Figure 7B) although not a perfect match.

## Discussion

We have shown that our AP generating model, the Paxon, can emulate an active AP passing through a spatial and temporal displacement that conforms to parameters similar to those of live biological sources, with the benefit of repeatability, stability in time, and programmability.

Variations in biological AP sources is normal. This variation is also what limits its application to testing and calibration of electrodes and supporting systems. The Paxon properties, albeit crude, open up new alternatives for both qualitative and quantitative assessments, from electrode to system, as well as variations between electrodes, compared to the biological sources (Searle and Kirkup, 2000; Kelly et al., 2007; Yoshida et al., 2009; Marozas et al., 2011) where only qualitative testing can be done. With the expected continuous development of new electrodes (Golestanirad et al., 2013; Meziane et al., 2013), electrode materials (Ravichandran et al., 2010; Pedrosa et al., 2012), and system configurations, quantitative methods of comparing these to each other in a standard and repeatable manner will be required.

A limitation to this model is that a single node does not present a wave shape similar to that of the Hodgkin and Huxley (1952) model, even though the composite signal over time does for standard surface Ag-AgCl electrodes (Figures 4A, 7B). The model in its current development represents a straight axon; branching or bends in a real axon are not modeled, nor are intervening tissues. Polynomial Equation (5) is only valid for the specific configuration used in this test case. The predictive polynomial equation may be refined in future designs to closer match the model's generated output. The flexibility of the generated AP could increase the model's application range to mimic a broader range of AP sources, and possibly non-healthy fiber function, e.g., demyelination. Other limitations include manufacturing processes which resulted in a fixed inter-nodal spacing of 2 mm implying a fixed propagation speed. A major challenge during the construction of the Paxon was in the preparation of the well, specifically with the application of the thin gold wire embedment in plastic resin for the building of the A-fibers mimic.

Smaller nerve structures, down to 1 μm, could have been developed using other manufacturing techniques such as wafer fabrication methods, or metal deposition and back-etching. However, the connectivity and interfacing with such smaller structures may present a challenge. The tradeoffs between axon diameters, node spacing and conduction velocity directed the choice of parameters for reasonably easy assembly with readily available materials for proof-of-concept.

In biological tissue, axons are not typically found as singular entities. Active tissues are always in a state of flux (e.g., a nerve at rest still leaks calcium, and the ion pumps are still working) so there are always variations in output signal with an AP event. Consideration of the complex biphasic distribution of axons ranging from fast-low threshold to slow-high threshold can lead to further refinements to the Paxon's AP emulation range. The intent with the Paxon is to simplify the source to a known set of values of expected amplitude and duration at the electrode face that can be applied and tested with. When comparing the ulnar nerve to the Paxon captured AP (Figure 4A, mean Paxon AP, and Figure 7B), there is a difference between the two. This difference can be minimized by adjusting the Paxon settings. The default values used were not specifically tuned to mimic this application.

The Paxon detected signals presented a positive maxima of 1.5 ± 0.05 mV including the 40x gain (Table 1) with a differential electrode pair positioned 10 mm above the nodes of Ranvier. Given the programmable nature of the driver (Figure 6), any drive potential between 0 and 2.8 V can be used. The relationship between the drive potential and the pulse width can be used to set the Paxon to target a desired AP process. Tune the “expected” output is programmed by applying Equation (5) as shown in Figure 6B.

The volume above the well floor (Figures 1C, 2C) is open to probing with a wide variety of electrodes such as traditional surface or needle electrodes (e.g., ECG, EMG, LIFE, TIME), as well as newer multi-pin contacts (e.g., Tathireddy et al., 2008). However, this model is not axially symmetric, which presents a problem for use with cuff electrodes. One recent “test verification” source for a cuff electrode is an earthworm (Yoshida et al., 2009), while this is functional it still relies on a biological source with its attendant problems. To test cuff electrodes, modifications to the Paxon set-up as well as testing strategy will be needed, e.g., wrapping the cuff around the model (Andreasen et al., 1997; Rieger et al., 2014). The Paxon as used for this proof-of-concept is also sensitive to some configuration variables, for example variations in the physical positioning above the well, and spacing of the discreet electrodes making up the differential pair.

The Paxon is not a complete biological axon equivalent as it lacks the detailed structures or function of an axon. Currently, the Paxon is limited to surface electrode probing. Patch clamp and similar electrode testing is not compatible with this model as there is no membrane to clamp to. There is sufficient flexibility within the Paxon design to possibly extend the model to include more complex scenarios. The Paxon model might also be used in conjunction with deep brain stimulation systems to find the coupling properties and transfer function of electrodes. This could help in the understanding of the electrode properties and to demonstrate the advantages and limitations of some of the recently proposed lead designs and stimulation modes (Hemm and Wårdell, 2010; Martens et al., 2011). New materials (Kip et al., 2011) and specialized coatings (Aregueta-Robles et al., 2014) being studied with regards to various implantable and surface electrode designs are another potential application area for the Paxon. The well design allows for some flexibility in electrode testing with future options to make a deeper, or longer axonal mimic. In all of these cases, the Paxon would serve as the AP generator, while the recording electrodes would be used to couple the electric field to instrumentation for data collection. Next steps in development could be to implement the Paxon concept in a microchip format or as printed electronics (Wang et al., 2012) thereby opening up the possibility to produce Paxons with different inter-nodal spacing and axon diameters, that can mimic a range of different nerve axons with much greater biological relevance. By redesigning the Paxon electronics and implementing a detector and regenerator circuit, the Paxon can be made to behave like active biological tissues with the saltatory nature of the myelinated axon. With this further development, it should also be possible to couple the Paxon to live tissues, as well as trigger the Paxon with devices such as a pacemaker system.

In conclusion, the Paxon is a novel programmable hardware design for the generation of AP signals. Repeatability, timing, and stability are some of the major advantages of the Paxon. The consistency of these Paxon parameters might permit its use as a reliable test platform for electrodes and measurement systems.

### Conflict of interest statement

The authors declare that the research was conducted in the absence of any commercial or financial relationships that could be construed as a potential conflict of interest.
